# Field evaluation of SumiLarv^™^ 2MR larvicide for controlling the invasive malaria vector *Anopheles stephensi* in Ethiopia: a randomised controlled trial

**DOI:** 10.1186/s12936-026-06089-3

**Published:** 2026-08-17

**Authors:** Alemayehu Dagne, Patricia Doumbe-Belisse, Teshome Degefa, Ahmed Zeynudin, Biniam Lukas, Adane Eyasu, Kasahun Eba, Fatou Jaiteh, Dereje Geleta, Ephrem Lejore, Ebisa Erena, Eba Alemayehu Simma, Koen Peeters Grietens, David Weetman, Martin J. Donnelly, Alison M. Reynolds, Anne L. Wilson, Delenasaw Yewhalaw

**Affiliations:** 1 School of Medical Laboratory Sciences, Institute of Health, Jimma University, Jimma, Ethiopia; 2 Tropical and Infectious Disease Research Centre, Jimma University, Jimma, Ethiopia; 3 Department of Vector Biology, Liverpool School of Tropical Medicine, Liverpool, UK; 4 Department of Environmental Health Sciences and Technology, Institute of Health, Jimma University, Jimma, Ethiopia; 5 Unit of Socio-Ecological Health Research, Department of Public Health, Institute of Tropical Medicine, Antwerp, Belgium; 6 School of Public Health, College of Medicine and Health Sciences, Hawassa University, Hawassa, Ethiopia; 7 College of Natural Sciences, Mada Wollabu University, Mada Wollabu, Ethiopia; 8 Department of Biology, College of Natural Sciences, Jimma University, Jimma, Ethiopia

**Keywords:** *Anopheles stephensi*, SumiLarv^™^ 2MR, Pyriproxyfen, Larval source management, Mosquito control, Emergence inhibition, Ethiopia

## Abstract

**Background:**

The establishment of the invasive malaria vector *Anopheles stephensi* in Ethiopia threatens existing malaria control efforts. Unlike native vectors, *An. stephensi* thrives in both urban and rural areas, lacks clear seasonal peaks of abundance, and often breeds in artificial water containers. SumiLarv^™^ 2MR containing the larvicide pyriproxyfen (PPF) offers a promising method for controlling the immature stages of *An. stephensi* in such habitats. This habitat-randomised controlled trial evaluated the efficacy of SumiLarv^™^ 2MR against *An. stephensi* in Danan town, Somali region, Ethiopia.

**Methods:**

Entomological surveillance identified 102 large artificial containers (“*birkas*”) which were randomly allocated in a 1:1 ratio to receive either SumiLarv^™^ 2MR (treatment arm) or no treatment (control arm). SumiLarv^™^ 2MR was introduced into the *birkas* at a dose of one disc per 200 L of water. Morphological identification was performed on adult mosquitoes emerging from control containers, while molecular analysis was conducted on both morphologically identified adult mosquitoes and dead pupae collected from the control and treatment arm. The residual efficacy of PPF content of the remaining discs was also analysed. Inhibition of adult mosquito emergence was assessed every 2 weeks for 40 weeks to determine the duration of efficacy.

**Results:**

The number of SumiLarv^™^ 2MR remained constant in the treatment arm throughout the study period. No significant differences were observed in water volume, pH, or temperature between the two study arms. Introduction of SumiLarv^™^ 2MR resulted in complete (100%) inhibition of adult emergence throughout the 40 weeks’ study period (t = 6.26, p < 0.05). Morphological identification of a sub-samples of adult mosquitoes emerging from control containers at different time points confirmed the presence of *An. stephensi* (99.85%) and *An. gambiae* s.l. (0.15%). Molecular analysis of morphologically identified adults from control containers and dead pupae from treatment containers confirmed that 98.91% were *An.stephensi*. Post-trial analysis showed that approximately 82.20% of the original PPF had been released from SumiLarv^™^ 2MR during study period.

**Conclusions:**

The high and sustained efficacy of SumiLarv^™^ 2MR demonstrated in this study suggests that it is a suitable tool for controlling *An. stephensi* in large artificial water storage containers.

**Supplementary Information:**

The online version contains supplementary material available at https://doi.org/10.1186/s12936-026-06089-3.

## Background

The expansion of the invasive malaria vector *An. stephensi* across Africa poses a significant threat to malaria control. Originating from South Asia and the Middle East, this mosquito species has rapidly colonized multiple countries in the Horn of Africa [1]. It is now widely and firmly established throughout Ethiopia [2–5] and has been implicated in increased local malaria transmission [6, 7].

In Africa, unlike most native malaria vectors, *An. stephensi* thrives in urban and peri-urban settings, often exploiting artificial water storage containers for breeding [8]. In addition, it has developed resistance to multiple insecticide classes used in vector control, including pyrethroids, organophosphates, and carbamates [9–11]. This broad-spectrum resistance to insecticides, coupled with outdoor resting and feeding preferences [12], raises concerns over whether core vector control interventions such as indoor residual spraying (IRS) and insecticide-treated mosquito nets (ITNs) may be insufficient to curb the spread of *An. stephensi* on the continent. Larval control targeting aquatic stages, by reducing the availability of suitable breeding habitats [13], is a promising tool for controlling the vector. Different larviciding strategies such as biolarvicides, insect growth regulators and larvivorous fish have been deployed against *An. stephensi* in its native range [14], but little is known about their effectiveness in Africa.

In the Somali region of eastern Ethiopia, large containers or “*birkas*” are commonly used as artificial water storage systems for domestic purposes, playing a crucial role in areas experiencing climate variability and scarce or limited access to surface or groundwater [15, 16]. Such artificial water containers are reported as preferred breeding sites for *An. stephensi,* supporting year-round proliferation and contribution to malaria transmission [6, 17, 18]. Targeting such large artificial water storage containers could therefore provide a sustainable and effective approach to reduce *An. stephensi* populations in urban (and peri-urban) areas.

SumiLarv^™^ 2MR is designed to provide long-lasting activity and represents a promising intervention for controlling mosquitoes that breed in stored water [19]. The product consists of small plastic discs impregnated with pyriproxyfen, an insect growth regulator that prevents the emergence of adult mosquitoes from pupae. SumiLarv^™^ 2MR was pre-qualified by the World Health Organization (WHO) following successful trials against *Aedes aegypti,* a major arbovirus disease vector [20].

Several studies have confirmed its residual efficacy under semi-field conditions in Brazil and Ethiopia [21, 22], however its effectiveness against *An. stephensi* in larger-scale field trials conducted under real-world conditions remains untested. The aim of this study was to evaluate the impact of SumiLarv^™^ 2MR on *An. stephensi* population in Danan town, Somali region, eastern Ethiopia.

## Material and methods

### Study area

**Fig. 1:**
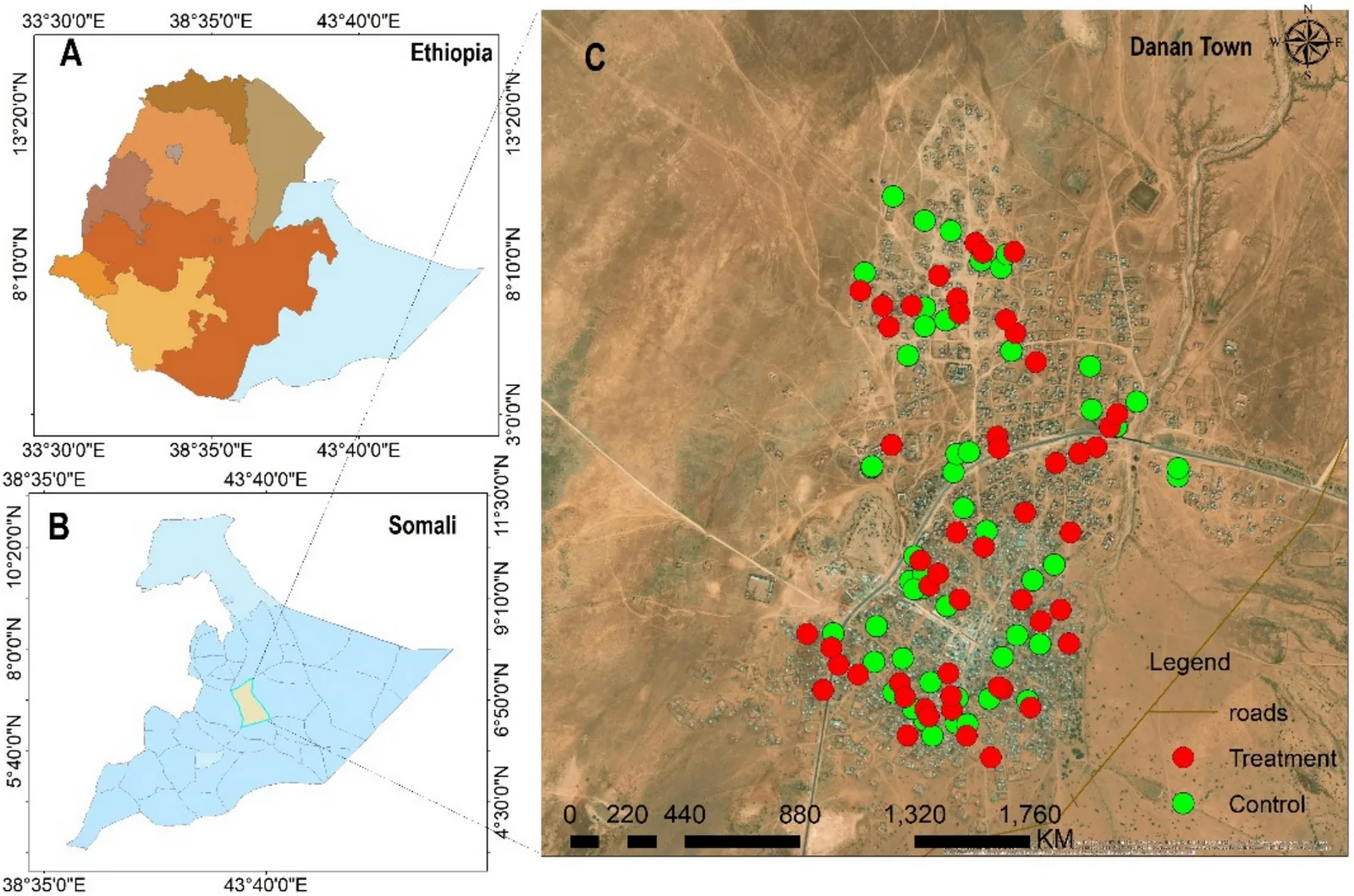
Maps of the study area (**A**) Location of Somali region within Ethiopia; (**B**) Location of Danan town within Somali region; (**C**) Location of treatment (red) and control (green) containers

Danan town (6.5131° N, 43.4958° E), located in the Somali region of eastern Ethiopia (the second largest region in Ethiopia) is approximately 1090 km east of Addis Ababa (Fig. 1). The region experiences a hot, arid climate with an average annual temperature of 28.4 °C and average annual rainfall of 19.24 mm [23]. The town consists of four administrative units called *“kebeles”* with an estimated overall population of 11,492 inhabitants. Malaria transmission is perennial, with *Plasmodium falciparum* and *Plasmodium vivax* as the main parasite species in the area, responsible for 60% and 40% of malaria cases, respectively [24]. The malaria control measures being implemented in the area are the use of insecticide-treated nets (ITNs) and Artemisinin-based combination therapy (ACT).

**Fig. 2:**
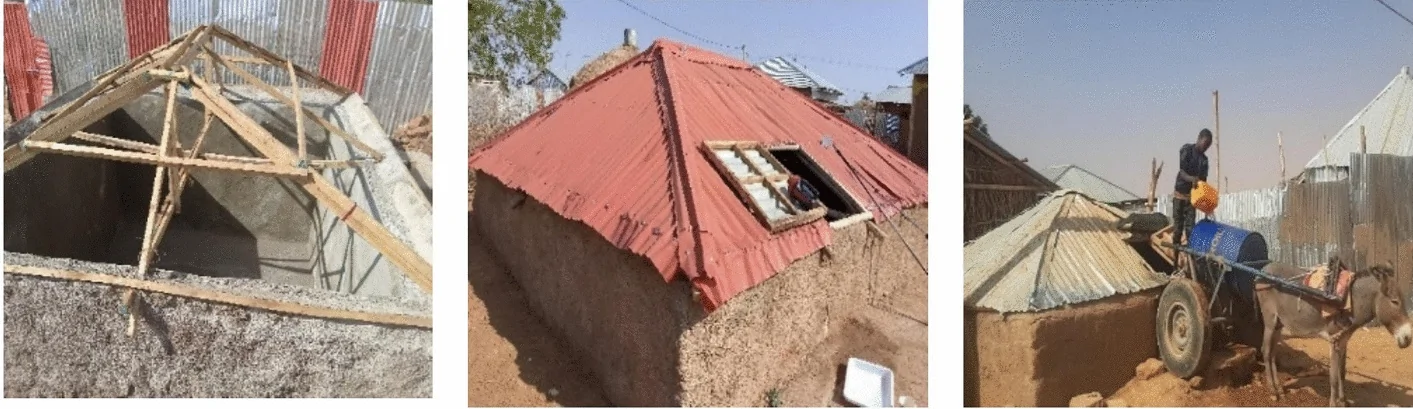
Photographs of large artificial water containers or *birkas* in Danan town, Somali region, Ethiopia

The population, predominantly of Somali ethnicity, relies on livestock rearing, small-scale trade, rain-fed sorghum farming, and remittances sent from abroad to support relatives and families. Recurrent droughts in recent years have led to food insecurity, livestock losses, and water shortages. Local houses are constructed from wood or concrete, with a variety of livestock kept within or immediate to the boundaries of the peridomestic space or compounds. River or rainwater is often stored in small plastic containers (barrels) for immediate household use and in larger concrete (20,000–28,000 L) corrugated-iron-roofed containers (*“birkas”*) with hatches for water removal and replenishment (Fig. 2). Water commercialisation by private individuals is a common practice for refilling *birkas,* costing households on average 13,000–15,000 ETB (approximately 100–125 USD, based on an exchange rate of 1 USB = 130 ETB) every 3–4 months. There are gender differences in water management at the compound level; women and girls majorly manage responsibilities related to water use and storage. The evaluation of the residual efficacy of SumiLarv^™^ 2MR against natural populations of *An. stephensi* was conducted in selected *birkas* across *kebeles* in Danan.

### Larvicide and dose rate

SumiLarv^™^ 2MR (Sumitomo Chemical Co., Ltd) is a polymer matrix release formulation housed within 5.5 cm diameter plastic discs, each weighing 2 g. The discs contain the insect growth regulator mimic, PPF at a concentration of 2% of the active ingredient (20 g AI/kg w/w). PPF inhibits the metamorphosis of mosquito pupae into adult mosquitoes. SumiLarv^™^ 2MR is designed for use in containers holding greater than 40 L of water, with low levels or absence of organic matter. This product is prequalified by WHO as a long-lasting larvicide for the treatment of artificial water containers against *Aedes* spp. and *An stephensi* [25]. The discs were stored under room temperature conditions in line with manufacturer recommendations until required.

### Study design

**Fig. 3:**
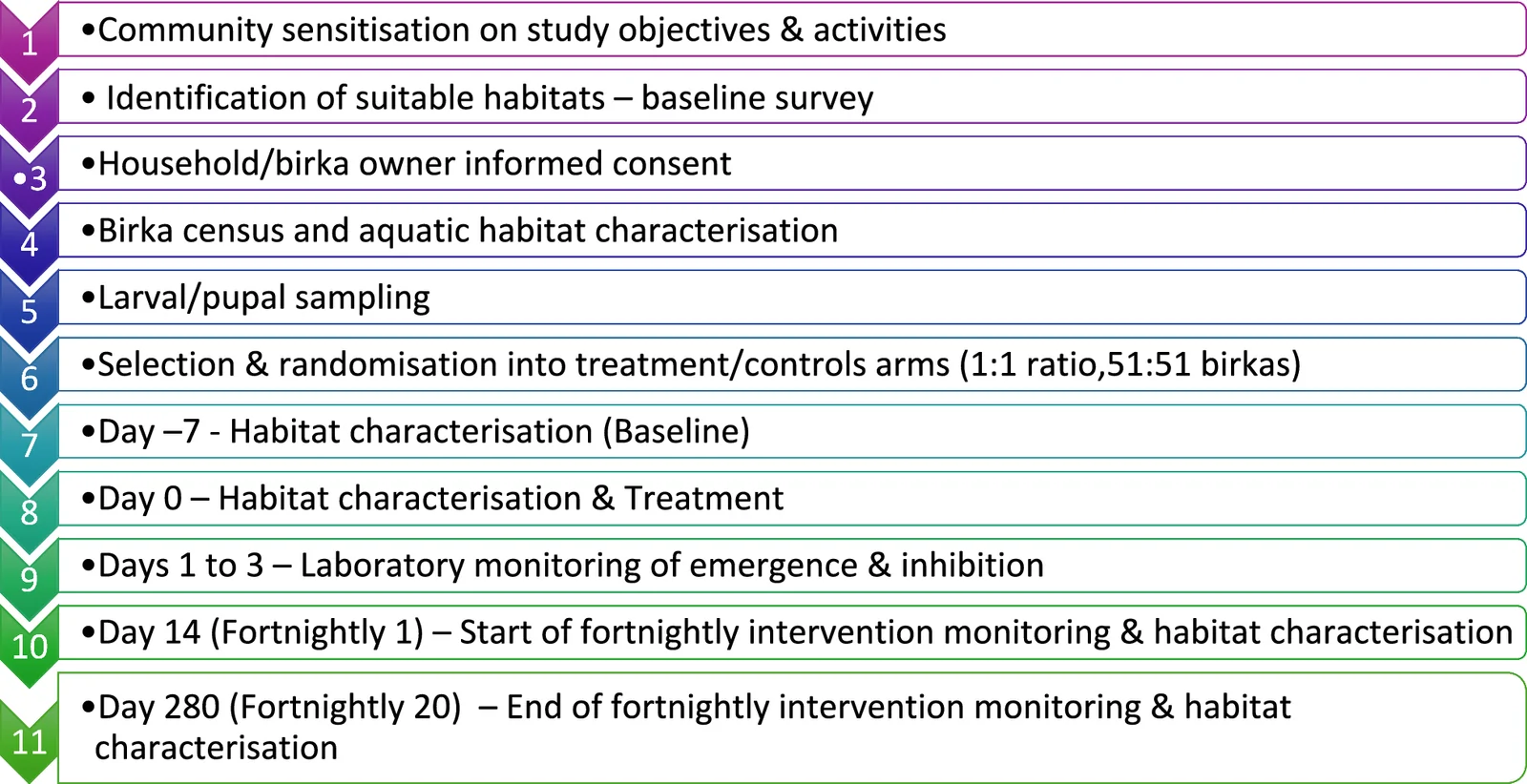
Workflow of the SumiLarv^™^ 2MR trial

This study followed the WHO guidelines for Phase III field studies [26]. Artificial breeding containers were randomised in a 1:1 ratio to receive either SumiLarv^™^ 2MR (treatment arm) or no treatment (control arm) (Fig. 3). The primary study endpoint was the inhibition of emergence rate (IE%) of *An. stephensi* between the two study arms, measured at the container level. A secondary endpoint was the determination of IE% for other mosquito species, including *Anopheles* spp. (e.g. *An. arabiensis*), *Aedes* spp. and *Culex* spp.

The trial was conducted between November 2023 and November 2024. A social science study was conducted alongside the trial to investigate community acceptability of the larvicides intervention and to evaluate the feasibility of engaging stakeholders in the development of localised implementation strategies. A separate manuscript describing these findings is in preparation.

Shortly before the implementation of the intervention social scientists engaged community members through participatory workshops aim at developing (i) tailored communication materials and health messages for community sensitisation campaigns and (ii) stakeholder engagement approach to facilitate regular dialogue regarding trial procedures and community perception. The health messages, and stakeholder engagement strategy developed during these workshops were subsequently implemented by the trial team before and throughout the intervention period.

### Community sensitization and stakeholder engagement

Prior to the intervention, community members and key stakeholders including (household owners, kebele officials, religious leaders, local health extension workers, district, zonal and regional health officers, and officials from the National Malaria Control Programme were engaged through a series of focus group discussions. Meetings were conducted by the Jimma University (JU) study team in collaboration with a Community Advisory Board (CAB) composed of locals community representatives and supported by social scientists from Jimma and Hawassa Universities. The purpose of these meetings was to introduce the study objectives and procedures and to obtain community approval for participation. Community approval was achieved through engagement with the CAB which acted as a representative body for the wide community prior to implementation of the study. Informed written consent was obtained from all adult participants. For participants with limited literacy, the purpose, procedures, risks, and benefits of the study were explained orally through community sensitization activities and house-to-house visits. Consent forms were provided in Somali, and a CAB representative was present as an independent witness to verify voluntary participation.

To improve awareness of the study and the potential benefits of the intervention, informative materials, including (posters), containing health messages developed by social scientist and researcher were produced in the local language (Somali) (Supplementary file 1). These materials were distributed throughout the community and directly to *birka* owners enrolled in the trial. They served as visual aids to reinforce key aspects of the study, malaria presentation, purpose and mode of action of the larvicide, its application, management, and safety concerns.

Household owners were also invited to participate in community meetings and those willing to take part in the study provided written informed consent. Community sensitisation activities and stakeholder dialogue regarding study progress and challenges faced continually discussed throughout the trial period. These activities were led by the Jimma University study team in collaboration with CAB and the District Health Officer.

Community acceptability was assessed through feedback provided by *birka* owners whose containers were treated with SumiLarv^™^ 2MR, and who subsequently shared their experiences with other community members. Acceptability was evaluated based on reported perceptions of the intervention arm expression of satisfaction and willingness to recommend the product to others.

### Data collection

#### Habitat census and physico-chemical characterization

Inspection and mapping of available artificial breeding habitats was conducted by a team consisting of Jimma University entomology technicians, locally trained data collectors, a district health officer, a malaria focal person and selected members of the CAB. The team surveyed all accessible artificial breeding sites across the town, including household and community water storage containers, plastic and steel drums, discarded vehicle tyres and larger concrete water storage containers. The geographic coordinates of each identified artificial container were recorded using a GPS mobile application [27]. All containers were checked for the presence of mosquito immature stages using long-handle dippers (350 ml capacity, BioQuip Products, Inc. California, USA), dipping through either of the two side-opening doors located at the front and back of the *birkas*, and sampling effort was standardized to 20 dips per container. Data recorded for each container included: habitat type, water type and usage, frequency of usage dimensions (width, breadth, depth), presence of a cover, presence and type of vegetation or algae, water volume (in litres) and presence/absence of predators. In addition, physicochemical parameters such as pH, temperature and turbidity were recorded using a Flintronic Digital high-accuracy pH tester (0.01 pH precision), a stainless-steel water resistance test thermometer (reference 258897) and a Palintest turbidity tube 26 (Model 1172427 5–500 NTU range) (Supplementary file 2). Larvae were counted and recorded regardless of their stage prior to disposal, while only pupae were reared to adult mosquitoes for morphological identification using the identification key developed by Coetzee et al. [28]. The abundance and diversity of mosquitoes were recorded in each container.

#### Duration of field exposure

SumiLarv^™^ 2MR discs were exposed under field conditions for duration of 40 weeks. (February 2024-November 2024). Containers were standardised based on their carrying capacity and volume. The mean daily temperature was 28.23 ± 1.73 °C with exposure to natural sunlight. Water renewal frequency occurred at 3–4-month interval based on household usage.

#### Selection of large artificial water containers, “birkas”

Based on the data recorded during the census, all *birkas* used to store water for domestic purposes including drinking (for both humans and animals), washing, construction, seedling irrigation, cooking and other household activities were identified. After the initial screening, immature mosquito stages were not present in all surveyed containers. Only artificial household water containers that were found positive for *Anopheles* spp. were re-surveyed for 7 days before treatment as a baseline assessment. We included in the study containers that were positive at –7 days from which we received the oral and written consent of the owners. They were then randomly assigned to the 2 study arms in a 1:1 ratio: the treatment arm received SumiLarv^™^ 2MR and the control arm received no intervention (Fig. 3).

#### Treatment: application of the larvicide to birkas

At the start of intervention, the SumiLarv^™^ 2MR were placed in *birkas* allocated to the treatment arm at a rate of 1 disc per 200 L of water according to the manufacturer's recommendations [19]. To calculate the required number of Sumilarv^™^ 2MR for each treated *birka*, 75% of the *birka* capacity was considered, as *birkas* were not normally full at day zero (T0). To prevent floating and possible removal, discs were tied together with rope and weighed down at each end with stones.

#### Evaluation of the SumiLarv^™^ 2MR efficacy

The primary endpoint was the emergence inhibition rate (IE%) of *An. stephensi* among pupae collected from treated *birkas*. To estimate the density of *An. stephensi* pupae in each *birka* throughout the study period, longitudinal entomological surveys were conducted 7 days prior to treatment (T-7), immediately before treatment (T0), 2 days post-treatment (T1), and subsequently every 2 weeks, alongside fortnightly habitat characterisation (Fig. 3). The trial was expected to continue until the emergence inhibition rate of the target species dropped below 80% at 2 consecutive residual time points.

At each subsequent time point, the same census parameters were recorded for each treated *birka*, together with the presence or absence of SumiLarv^™^ 2MR. Immature mosquito stages were collected using long-handled dippers (350 ml capacity, BioQuip Products, Inc. California, USA). Larvae collected during sampling were counted, recorded and discarded, whereas pupae, together with water from corresponding *birka*, were transported to the field insectary and monitored for adult emergence over a 72 h’ periods.

#### Residual PPF analysis in SumiLarv^™^ 2MR retrieved from field

Following completion of the 40-week trial, SumiLarv^™^ 2MR were retrieved from *birkas* in the treatment arm. To reduce analytical costs, residual PPF content was analysed in a sub-sample of discs only. For this purpose, discs were randomly selected from 8 *birkas* with the largest water volumes and 8 *birkas* with the smallest water volumes at week 40**.** Each selected disc was wrapped in aluminium foil, labelled, and transported to the Vector Health International Africa Technical Research Centre (ATRC), Tanzania, for chemical analysis to quantify the remaining PPF content.

#### Analytical technique and method validation

The method was carried out according to published Collaborative International Pesticides Analytical Council (CIPAC) procedure [29] for determining the residual active ingredient PPF content in the used SumiLarv^™^ 2MR. The materials used were PPF technical grade (Sumitomo Chemical Co., Ltd., Lot No.120109, Purity 99.2%), Dicyclohexyl Phthalate (Sigma-Aldrich, Lot N0. 08122TVD), Ethyl acetate (Loba Chemie PVT Ltd), Lot N0. B350522106, SumiLarv^™^ 2MR unused samples (Lot No. 2X01MRG), SumiLarv^™^ 2MR used in field trial (Lot No. 1Z01MRW).

Percentage recovery was evaluated using fresh SumiLarv ^™^ 2MR from the ATRC stock with a measured PPF content of 1.98% w/w. Linearity of the analytical method was assessed using PPF standards prepared at three different concentrations (0.5, 1.0, and 2.0 mg/mL) (Fig. 1, Supplementary File 3). Limit of detection (LOD), limit of quantification (LOQ), and intra- and inter-assay precision were not generated as the study was designed to answer specific scientific questions using a previously validated CIPAC method for comparative analysis of the study (Supplementary File 3) rather than meet regulatory validation requirements.

#### Preparation of standard solutions

Internal standard solution was prepared by dissolving 2.50 g of dicyclohexyl phthalate in 200 mL of ethyl acetate. This solution was further diluted tenfold to make a sufficient volume for sample extraction. PPF calibration standards (CA & CB) were prepared in duplicate by accurately weighing between 45 and 55 mg of PPF and dissolving it in 90.0 mL of ethyl acetate. An aliquot of 10.0 mL of the internal standard solution was then added to each calibration solution.

#### Extraction protocol of SumiLarv^™^ 2MR

Unused samples were prepared for this analysis by cutting approximately 0.5 g of disc into pieces, placing them in a 20 ml vial, adding 10.0 ml of the internal standard solution, and allowing the mixture to stand with shaking for 24 h at room temperature. This was followed by filtration and analysis using reversed phase high performance liquid chromatography (HPLC 1260 Infinity from Agilent Technologies) with UV detection at 254 nm and dicyclohexyl phthalate as the internal standard. Used SumiLarv^™^ 2MR was treated similarly, using approximately 1.0 g of material. The sample where cut into small pieces and 10.0 ml internal standard solution, followed by extraction at room temperature, filtration, and HPLC analysis using UV detection at 254 nm after method verification with an ATRC unused sample.

The detailed methodology, including materials, preparation of standard solutions, extraction procedures and HPLC analysis, is provided in supplementary file 3.

### Mosquito preservation and species identification

At each fortnightly data collection time point, pupae from both treatment and control arms were monitored for emergence in the temporary field insectary for up to four days. Pupae were placed in mesh-covered paper cups filled with water from their respective *birkas.*

Adult *Anopheles* mosquitoes emerging from control arm pupae were collected daily using mouth aspirators from labelled paper cups according to the date of collection and *birka* ID. These specimens were then transferred to new, mesh-covered, pre-labelled paper cups for temporary holding. Mosquitoes were immobilized using chloroform vapour and subsequently sorted by genus and sex. Species-level identification was performed using standard morphological identification keys [28]. Specimens identified as *An. stephensi* were placed individually in 1.5 ml Eppendorf tubes with silica gel, and retained for further molecular identification.

Pupae that failed to emerge (i.e., dead) from all the *birkas* were collected daily from the paper cups using pipettes and transferred to Whatman filter paper No. 1, for morphological identification of Anophelinae and Culicinae to genus level. They were then air-dried and transferred using forceps to individual 1.5 ml Eppendorf tubes containing 96% ethanol and immediately preserved at − 4 °C for further molecular identification.

### Molecular identification of immature and adult mosquitoes

DNA was extracted from mosquito wings and legs or preserved dead pupae following the Musapa et al. protocol [30]. A TaqMan-probe-based multiplex qPCR was performed for molecular identification of different species [31–33]. The control samples used for this analysis came initially from optimized templates of the morphologically identified, known adults from wild *An.stephensi* and susceptible *An. arabiensis.*

For one reaction, the master mix consisted of 6 μL TaqMan^™^ Fast Advanced Master Mix (Applied Biosystems), 0.4 μL each of stq-F and stq-R primers, with 0.5 μL FAM probe targeting *An. stephensi*, 0.4 μL each of AaAg-F and AaAg-R primers with 0.5 μL VIC probe targeting *An. arabiensis*, and 1.4-μL nuclease-free PCR-grade water. 10 μl of the master mix was transferred into each well of the 96-well PCR plate, and 2 μl of template DNA was added to each well and run on a Quant Studio^™^ 3 Real-Time PCR instrument (Applied Biosystems by Thermo Fisher Scientific 96- Well 0.2 mL Block).

The last four wells were controls: [1] FAM-positive control *An. arabiensis* colony (Sokoru susceptible strain), (2) VIC-positive control (confirmed *An. stephensi* DNA), (3) negative control (water) and (4) blank (no sample).

The plates were then sealed. The qPCR cycling conditions were set as follows: initial steps at 50 °C for 2 min, followed by 95 °C for 2 min, and then for 45 cycles at 95 °C for 3 s and annealing/extension at 60 °C for 30 s, for a total running time of 1 h and 6 min. PCR outputs were then analysed using Quant Studio design & analysis software v1.3.1.

### Data management and analysis

Data collected throughout the study were uploaded to the REDCap electronic data capture system [34]. Prior to analysis, data was downloaded, cleaned in Microsoft Excel and converted to CSV format for further analysis using R software [35]. Census data were summarised using descriptive statistics, including the proportion of *birkas* positive for *Anopheles* spp. and *An. stephensi* and as well as measure of productivity, expressed as (mean number of larvae and pupae per *birka*).

The number of discs considered according to the capacity of each treated *birka* was calculated as follows:

$${\text{Number of discs }} = \frac{1}{200}{\times}\frac{75}{{100}}\text{ capacity of birka}\times  \mathrm{Width} \times  \mathrm{Breadth} \times  \mathrm{length} \times 1000 $$

Adult Inhibition Emergence Rates (IE%) were compared between arms and estimated using Mulla’s formula [36]:

$$\mathrm{EI}(\%) = 100 - \left(\frac{T \times 100}{C}\right)$$

where T and C are the number of adult mosquitoes emerging from treatment and control arms, respectively.

Variables such as temperature, pH, and the mean number of pupae were compared using *t*-tests.

A generalised linear mixed model (GLMM) fitted with a negative binomial distribution (nbinom2) and a log link function were used to assess the relationship between the number of pupae (response variable) and the treatment arm (fixed effect). The habitat ID (habitat number at individual level) was considered as a random effect. The analysis was carried out with R.4.4.1 software [35] using the packages dplyr, ggplot2, tidyverse, tidyr, lme4, glmmTMB.

One-way ANOVA was used to evaluate PPF variability within strings and between containers.

### Selection of sub-sample for molecular analysis

As no prior molecular species identification data were available, the sample size was estimated using the single population proportion formula (unadjusted). A proportion of 50% (maximum variance), a 95% confidence level (z = 1.96), and a 5% margin of error were assumed, resulting in an initial sample size of 384. As the target population was finite, known and relatively small (N = 3881) a finite population correction was applied resulting in a required sample size of 388, after allowing for 10% a failure rate. The calculated sample was allocated equally between the control and treatment arms (194 specimens per arm). A stratified random sampling approach was used, with strata defined by study arm (treatment versus control) and time point (census to week 40). Within each stratum, a 10% random sub-sample was selected using the true random number generator [37]. Of the selected specimens 10 from each study arm could not be analysed due to shortage of reagents and primers.

## Results

### Physico-chemical characterization of selected birkas

**Fig. 4:**
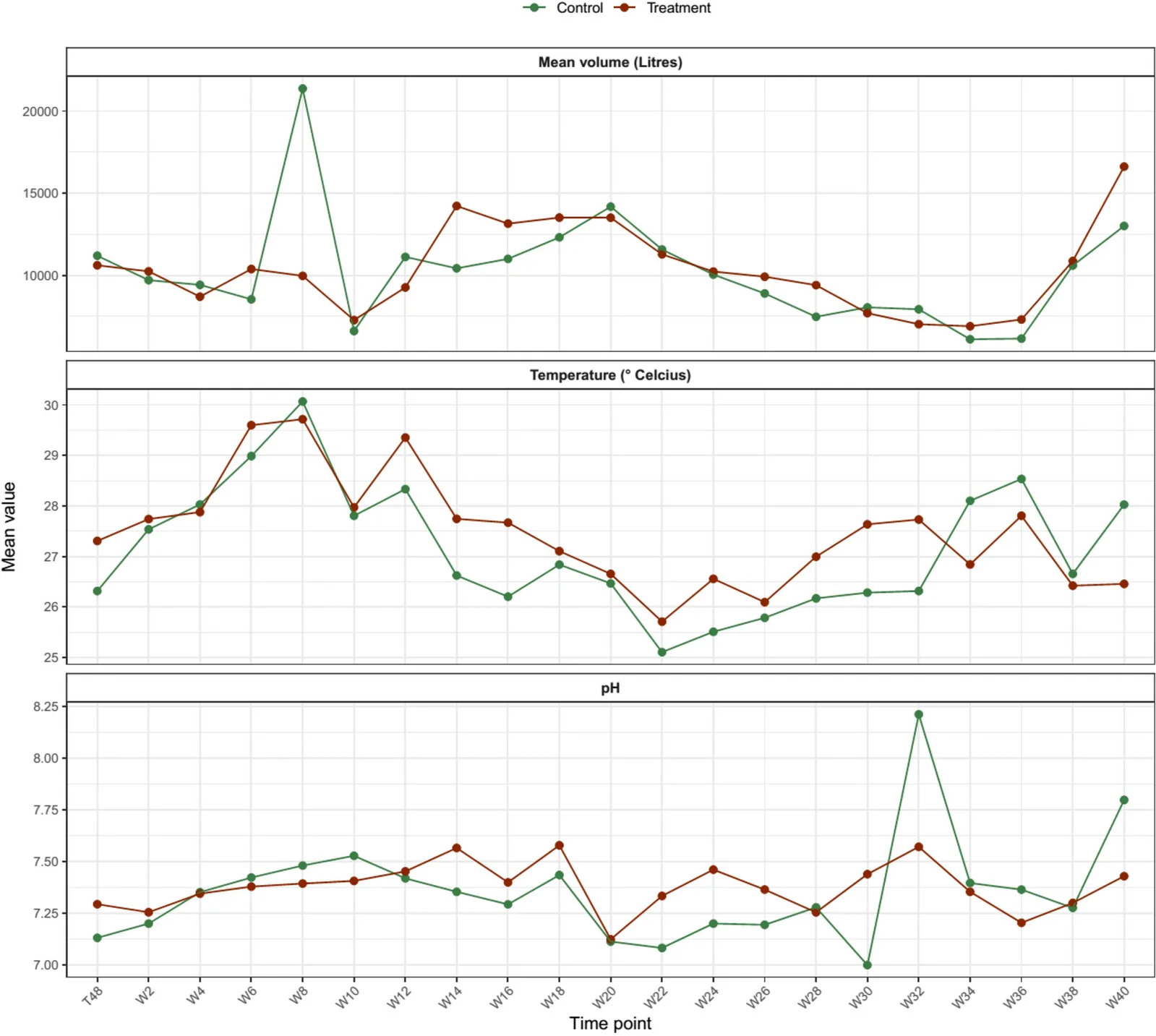
Mean water volume, temperature and pH of water in artificial containers. *Green line: control arm containers, blue line: treatment arm containers*

A total of 102 *birkas* were included in the study. The mean water volume in these *birkas* varied across time points due to regular domestic consumption and ranged from 6115 to 21,374 L (Fig. 4). There was no significant difference in mean water volume per habitat between the two arms (t = − 0.12, p = 0.90). The mean water renewal frequency during the study period was 3.05 ± 0.88 (95% CI 2.88–3.22), usage varied according to family size. There was no significant difference in water renewal frequency between the two arms (t = 1.24, p = 0.66).

During the study water temperature ranged from 25.11 to 30.07 °C and pH levels varied between 7.0 and 8.2 (Fig. 4). No significant differences were observed between the treatment and control arms in temperature (t = − 0.94, p = 0.35) or pH (t = − 0.27, p = 0.78).

### Treatment of birkas using SumiLarv^TM^ 2MR discs

A total of 5075 Sumilarv^™^ 2MR discs were introduced into 51 treatment *birkas* (Supplementary file 4). The mean number of discs introduced into each *birka* initially was 99.5 (Supplementary file 5). Disc retention was 100% until week 36, at which time 54 discs were lost from one *birka*.

### Birka positivity for anopheles

**Fig. 5:**
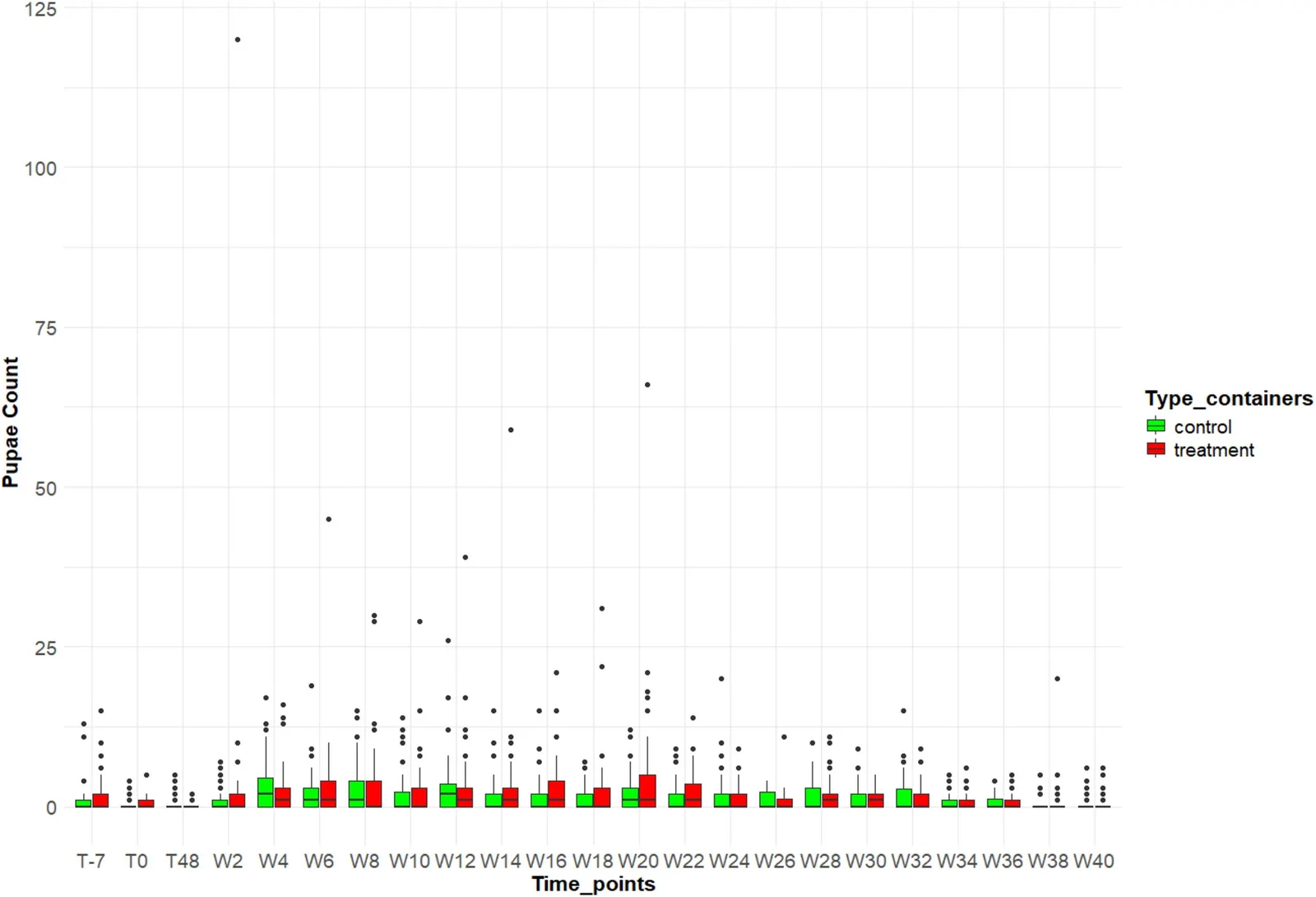
Distribution of mean number of pupae in study arms across time points

Over the study period, 1831 and 2050 pupae were collected from treated and control *birkas*, respectively. There was no significant difference in the mean number of pupae collected across time points between treatment and control arms (p = 0.111) (Supplementary 6; Fig. 5).

Of the sample of 2050 adult mosquitoes reared from pupae collected from control *birkas* (from the census period to the end of the study)*. An. stephensi* accounted for 99.85% (95% CI, 99.57–99.95; n = 2047) while *An. gambiae* s.l accounted for 0.15% (95% CI, 0.05–0.43; n = 3). From a random selection of 184 mosquito samples from the control *birkas* 182 (98.91%) were confirmed to be *An. stephensi* via qPCR screening while the remaining 2 (1.09%) could not be identified, validating the results of the initial morphological identification (Supplementary file 7). Other vector species, including *Aedes* and *Culex*, which are commonly associated with artificial containers, were not observed. A subsample of 184 dead pupae from the treatment *birkas* was analysed for molecular identification and species confirmation. Of these, 182 (98.91%) were confirmed as *An. stephensi*, while the remaining 2 (1.09%) could not be identified (Supplementary file 7).

### Inhibition emergence rates in treatment and control arms

**Fig. 6:**
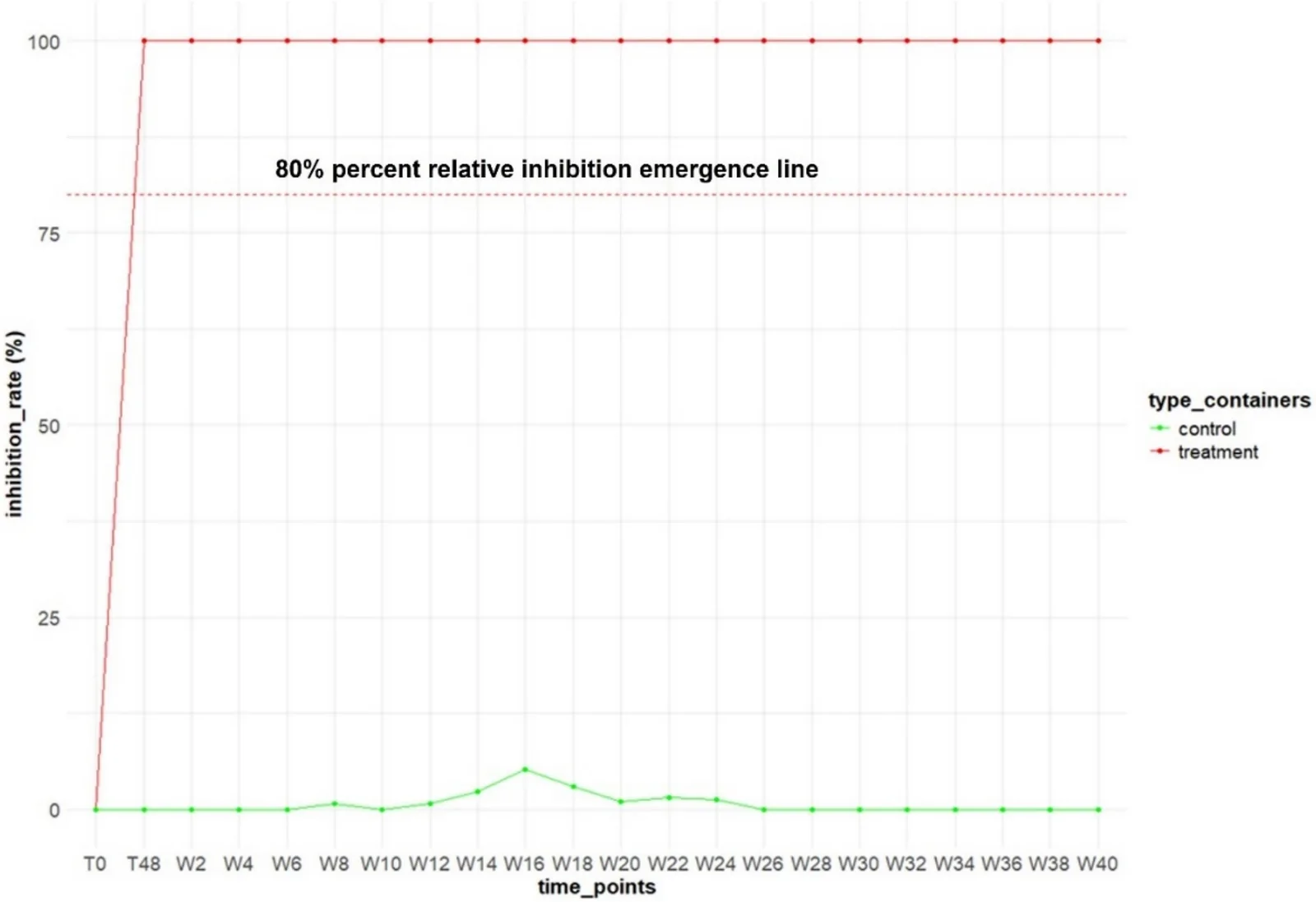
Emergence inhibition rates (EI%) of *Anopheles* pupae collected from different types of large artificial containers or *birkas*

At baseline (T0), the emergence inhibition rate in both study arms were zero. Two days after the treatment, the inhibition emergence rate in the treatment arm was 100% and remained the same until the end of the study (week 40). In the control arm, EI% varied from 0 to 6% throughout the study period (Fig. 6). The difference between intervention and control arms in EI% was highly significant at all-time points (t = 6.26, p = 1.5e-07).

### ***Field residual results of PPF content analysis in Sumilarv***^™^*** 2MR discs***

A total of 48 SumiLarv^™^ 2MR samples from 16 selected containers (3 samples were collected in each container; 2 from the ends of the string + 1 from the middle).

Method validation indicated the calibration curve exhibited excellent linearity, with a correlation coefficient (R^2^) = 1.000 (Fig. 3, Supplementary file 3). In addition, the mean percentage recovery of PPF content from fresh SumiLarv^™^TM 2MR (1.98%w/w) was 99.00%, indicating efficient extraction of the active ingredient.

The average PPF content in the fresh sample (2X01MRG) from ATRC stock, after confirming the analytical method (Supplementary file 3), was 1.98%w/w, closely aligning with the expected reference value of 2.0 ± 0.5%w/w. The estimated 82.10% release was calculated based on an initial concentration of 1.98%w/w with the mean 0.36 ± 0.20% w/w (95% CI: 29.63–0.41.33%) residual concentration of field collected Sumilarv^™^ 2MR. The uncertainty around the estimated release was 82.10% ± 3.0%, corresponding to arrange of 79.1–85.1%)(Supplementary file 3 and 10).

**Fig. 7:**
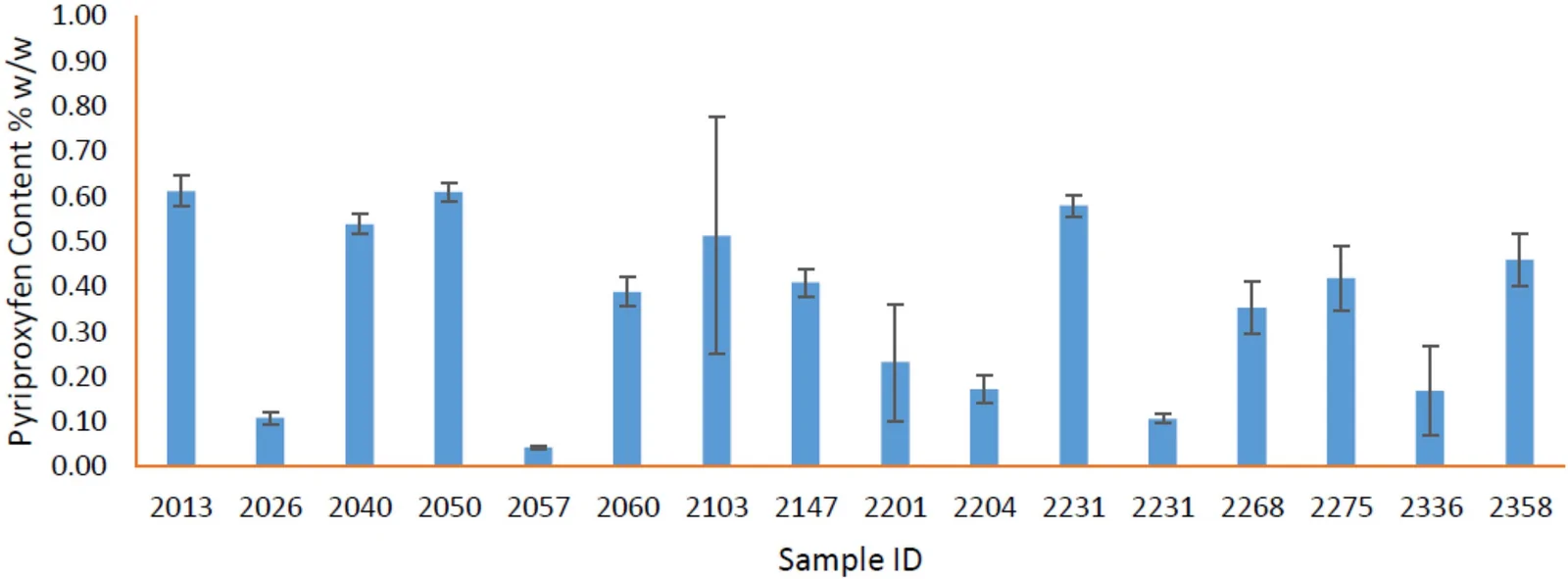
PPF content in Sumilarv^™^ 2MR after 40 weeks in artificial containers in Danan town, Somali region, eastern Ethiopia. Blue bar represents the mean value of three independent samples (n = 3)

The lowest PPF content was observed in discs from sample ID 2057, all three sub-samples (Middle (M), end1, end2) contained only 0.04 ± 0.00% w/w (95% CI: 0.004–0.004%). The highest PPF content was found in sample ID 2013, which showed an average PPF content of 0.61 ± 0.03%w/w (95% CI: 0.52–0.70%) across its three sections (Fig. 7).

Notably, certain samples exhibited a significant variation between sub-samples of discs tied together with a string, indicating potential uneven release of the insecticide. For example, sample ID 2103 showed values ranging from 0.21%w/w to 0.68%w/w, while sample ID 2336 fluctuated between 0.10% w/w and 0.28% w/w (Fig. 7). Particularly, sample ID 02026 and ID 02231 consistently showed low PPF levels (as low as 0.10%w/w).

Overall, the results suggest that while PPF remains detectable in used discs, the average remaining PPF content across all samples was approximately 0.36% indicating about 82.10% of the original concentration was released during the trial period (supplementary files 8, 9).

### Variability analysis

The coefficient of variation (%CV) for the sub-samples is provided in supplementary file 10.

One-way ANOVA was used to evaluate PPF variability within strings and between containers. The result indicated no significant difference between the middle and end sections of the strings (F = 0.000005, p = 0.998), suggesting homogenous distribution of PPF along the string length. In contrast, significant differences were observed among containers (F = 16.13, p < 0.001), demonstrating substantial inter-container variability.

## Discussion

Targeting artificial larval habitats could be an effective strategy for reducing or suppressing *An.stephensi* populations within its invasive range, given its ability to persist year-round in these sites [38].

In this trial in Danan, emergence inhibition rates were 100% in the treatment arm over the 40-week follow-up period. This clearly demonstrates that SumiLarv^™^ 2MR is effective at a dosage of one disc per 200 L of water against *An. stephensi* in large artificial water storage containers in our setting. Studies in Brazil [21], Cambodia [39] and Myanmar [40] also demonstrated the effectiveness of SumiLarv^™^ 2MR in controlling *Aedes* mosquitoes, which also typically breed in artificial containers. In Brazil, SumiLarv^™^ 2MR showed sustained efficacy of over 80% emergence inhibition for approximately 60 weeks against *Ae. aegypti* [21]. Similarly, studies conducted in Myanmar showed that this larvicide remained effective for at least 6 months, with *Aedes* sp. adult mosquito emergence in treated water remaining below 20%, compared to over 90% in untreated water [40].

Water volume in the study containers varied over time due to daily household usage, periodic replenishment from local rivers, water purchases from private suppliers, rainwater harvesting during the rainy season. However, water availability from these sources was strongly influenced by the climate variability and limited, irregular rainfall, which is typically concentrated in two short rainy seasons [41]. These climatic conditions often led to inconsistent water replenishment, affecting the volume and frequency of water stored in the containers. To manage those shortages, participant communities also relied on water from the distant Wabi-Shebele River located approximately 80 km from Danan town and accessed through commercial vendors who transported it by truck at a substantial cost.

While such fluctuations have been reported to interfere with the residual efficacy of other larvicides used for the control of outdoor mosquito species [42], our data showed no evidence that variation in water volume affected the efficacy of SumiLarv^™^ 2MR. This is in line with previous reports in Cambodia showing that water replacement or removal did not affect the efficacy of SumiLarv^™^ 2MR against *Ae. aegypti*. In treated domestic water jars, the emergence inhibition rate remained above 90% for the first 20 weeks and above 80% until the study concluded at 34 weeks. PPF remained effective despite regular water usage and refilling of the jars [39]. It is worth highlighting that the replacement of water in large containers, such as *birkas*, is often less frequent than in smaller containers, which are also more prone to human disturbances [40], limiting any disc loss and maintaining residual efficacy in large containers. Adaptations to water scarcity, including the high costs associated with refilling *birkas* (13,000–15,000 ETB or 100–125 USD approximately per delivery by compound), tend to limit water usage and promote prolonged storage. This practice creates opportunities for the implementation of control measures such as SumiLarv^™^ 2MR.

A more limited residual efficacy of SumiLarv^™^ 2MR was observed in a semi-field trial conducted in Ethiopia using small artificial plastic containers (barrels) which included 50% water replacement on a fortnightly basis. The authors reported 100% emergence of inhibition for 29 weeks in the 250 L water containers treated with one Sumilarv^™^ 2MR, which then declined to 93% by week 35 of the treatment [22]. In contrast, the current trial reported 100% emergence of inhibition for up to 40 weeks. This may partially be explained by the fact that the mean number of discs remained very stable, with 100% retention recorded for 36 weeks and remaining residues between refills in the treatment *birkas* and the sustained release of PPF from the discs throughout the study. This effectiveness is further supported by the analysis of the remaining PPF content, indicating about 83% of the original concentration released during the trial period.

Residual efficacy was maintained in the discs even when only 0.36% of the active ingredient remained, indicating that the formulation can continue to exert biological activity at very low concentrations. This is consistent with published findings from Darriet et al. [43] laboratory evaluation who reported that PPF remains highly effective at very low ppb (parts per billion) concentrations, with substantial adult emergence inhibition observed in *Ae. aegypti as low* 3.3 × 10^–4^ mg/L. (0.33 ppb). Similarly, findings from Iquitos, Peru [44] showed that PPF prevented emergence with LC50 values around 0.012 ppb demonstrating high sensitivity of mosquito larvae to the compound. These findings suggest that PPF can achieve biologically meaningful effects at very low concentrations, even when only a small fraction of the original product remains, supporting its potential for long-lasting efficacy.

The high release rate of PPF may be influenced by the water temperature of containers, as warmer temperatures are known to accelerate chemical processes [45]. Although temperature can impact release dynamics, some studies report that the SumiLarv^™^ 2MR release technology is designed to maintain a consistent PPF concentration in containers [22]. The absence of a significant difference between the middle and the two end sections of the individual discs indicates that PPF release was generally uniform along each string with only minor variation in a few sub-samples. This consistent release across the three discs’ sections is likely attributed to surface erosion acting uniformly on all exposed surfaces under similar temperature conditions. In contrast, significant inter-container variability was observed (F = 16.13, p < 0.001). This variability may be explained by external environmental factors such as hydrolytic degradation of the discs, differences in water turnover rates leading to variable leaching, manufacturing variability, and potential biofilm formation among containers. Additionally, water quality may also be considered as a contributing factor.

Entomological monitoring during the trial showed no significant difference in the mean number of pupae between the treatment and control arms, despite significant differences in emergence inhibition rates. This suggests that PPF prevents adult emergence without reducing the density of larvae and pupae, consistent with findings from a previous study in Cambodia [39]. Species identification confirmed the predominance of *An. stephensi*, followed by *An. gambiae* s.l. The remaining, 2 could not be identified from each arms were indicated no amplification (undetermined cycle threshold value). This may be due to pipetting errors (no DNA added), degraded DNA or presence of substances that inhibit PCR. Other vector species, such as *Aede* spp. and *Culex* spp. which are typically found in artificial containers, were not detected. This underscores the importance of considering species-specific ecological preferences [46]. *Aedes* spp. mosquitoes for instance, tend to favour medium-sized (10–200 L) containers while *Culex* spp. mosquitoes prefer organically enriched containers with darker coloured water [47]. In contrast, the larger artificial containers used in this study may have been less suitable for their oviposition and larval development. This divergence in habitat selection implies that interventions optimised for *Aedes* may not automatically translate to effective control of *An. stephensi* even within the same urban setting.

Understanding the bioecology of different mosquitoes’ species is essential before selecting and implementing any control method [48]. The reported presence of fish (*Oreochromis niloticus*), frogs, and toads during the monitoring in both study arms suggests that the product does not affect non-target organisms. Although previous studies have reported potential adverse effect of PPF on the aquatic ecosystem [49], the lack of local data limits our ability to draw conclusions about its ecological safety in Ethiopian settings.

Community acceptability of the product is important for the widespread implementation of this larvicide product for controlling the invasive vector *An. stephensi* at national level. SumiLarv^™^ 2MR offer operational advantages over other larval control products being easy to handle and providing long-lasting residual efficacy. Good acceptance was also reported in Cambodia following the engagement of health volunteers prior to the study [50].

The study’s intervention delivery approach was informed by the community’s socio-political structures and concerns regarding the product, ensuring meaningful stakeholder involvement through interactive dialogue and sensitisation through tailored communication material and health messages. This approach highlights the critical role of community engagement and participatory research methodologies in aligning community expectations with project objectives during the planning and implementation of vector control interventions.

To effectively incorporate SumiLarv^™^ 2MR into malaria vector control programmes, national health authorities should prioritise its adoption for treating long-term water storage containers particularly in urban and peri-urban settings where long-term water storage is common due to water scarcity. Community health workers or trained local volunteers could be mobilized to maximise impact [51]. Application could be done every 6 months, aligning with the product’s long-lasting efficacy. However, further operational research is needed to optimize deployment strategies, monitor coverage, and assess any emergence of insecticide resistance. Ongoing surveillance should also evaluate the impact of climate variability and urban water storage practices on *An. stephensi* breeding patterns to ensure adaptive, targeted use of SumiLarv^™^ 2MR.

## Limitations

This study has several limitations. First, entomological monitoring was not continued until emergence inhibition dropped to 80%, due to resource constraints. As a result, it was not possible to determine the duration of efficacy beyond the 40-week study period or to recommend an evidence-based retreatment interval.

A second limitation relates to the analytical method used to quantify PPF release from Sumilarv^TM^ 2MR discs. The assay was developed as a fit-for research purpose, not to develop or validate a new analytical method. Therefore, parameters such as the limit of detection (LOD), limit of quantification (LOQ), intra and inter-assay precision, or measurement uncertainty were not generated. Although all analyses were performed under controlled conditions and considered suitable for comparative analysis within this study, the lack of full validation limits direct comparison of absolute values with those generated using fully validated analytical methods.

## Conclusions

This study provides strong evidence that SumiLarv^™^ 2MR is a promising tool for controlling immature *An. stephensi* populations within its invasive range, owing to its long-lasting efficacy and ease of application. Further action is needed at the national level to drive policy change and to facilitate the incorporation of Sumilarv ^™^ 2MR into local integrated vector control strategies. Such efforts could help protect vulnerable populations from this invasive malaria vector in urban and peri-urban areas of Ethiopia, as well as other affected regions with similar environmental conditions, including climate variability, water scarcity and a reliance on long-term water storage in large artificial containers.

## Supplementary Information


Additional file 1Additional file 2Additional file 3Additional file 4Additional file 5Additional file 6Additional file 7Additional file 8Additional file 9Additional file 10Additional file 11

## Data Availability

No datasets were generated or analysed during the current study.
