# Supplementary material for: Field evaluation of SumiLarv™ 2MR larvicide for controlling the invasive malaria vector Anopheles stephensi in Ethiopia: a randomised controlled trial
Source: Malar J. 2026 Aug 17;25:314. doi: 10.1186/s12936-026-06089-3 (PMC13531892; doi:10.1186/s12936-026-06089-3)
Supplement: Supplementary file 1 [file 12936_2026_6089_MOESM3_ESM.pdf]

CEASE Form 3b-1 Parts 3-7 Longitudinal Habitat Census Characterisation

Please complete the survey below.

Thank you!

PART III: Habitat Characteristics

Start time of habitat census

____
((hours:minutes, use 24h clock e.g. 9.00pm = 21.00))

Finish time of habitat census

____
((hours:minutes, use 24h clock e.g. 9.00pm = 21.00))

Habitat width (specify in metres)

____

Habitat length (specify in metres)

____

Habitat depth (specify in metres)

____

Estimated habitat volume at the time of the survey (specify in litres)

____

Does the habitat have a cover?

- ◯ No
- ◯ Yes, partially covered
- ◯ Yes, fully covered

How often is the water used?

- ◯ No use
- ◯ At least daily
- ◯ Every other day
- ◯ Weekly
- ◯ Less frequently than weekly

PART IV: Water characteristics

Is there algae in the habitat?

- ◯ None
- ◯ Scarce
- ◯ Moderate
- ◯ Abundant

What type of algae is present in the habitat? (select all that apply)

- ◯ Filamentous
- ◯ Green
- ◯ Blue Green
- ◯ Brown
- ◯ Unidentified

Is there vegetation (excluding algae) present in the habitat? (select all that apply)	◯ None<br>◯ Scarce<br>◯ Moderate<br>◯ Abundant
What type of vegetation (excluding algae) present in the habitat? (select all that apply)	◯ Submerged<br>◯ Floating<br>◯ Emergent<br>◯ Other ______
Is there refuse/visible waste in the habitat?	◯ Yes<br>◯ No
Is there other surface film in the habitat?	◯ Yes<br>◯ No
Is there suspended soil or dust in the habitat?	◯ Yes<br>◯ No
Are there predators visible in the habitat?	◯ Yes<br>◯ No
If yes, what type of predators?	◯ Fish<br>◯ Tadpole (frog larvae)<br>◯ Invertebrate<br>◯ Other ______
Has the water been treated with any chemical/s?	◯ Yes<br>◯ No
If yes, what type of chemical?	◯ Chlorine<br>◯ Oil<br>◯ Other {main_water_chem_type_oth}
Other chemical, please specify.	________________________________
Is the habitat in the shade?	◯ No<br>◯ Yes, partial shade<br>◯ Yes, full shade
Record the temperature of the water	________________________________<br>(Record in degrees celcius)
Record the pH of the water	________________________________<br>(Record from 1 to 14)
What is the turbidity value of the water?	________________________________<br>(Read from the turbidity tube)

PART V: Larval sampling

Have you taken a photo of the habitat?	◯ Yes<br>◯ No

If yes, please ensure photos are stored referencing Habitat ID number (from Part I)

Please upload photo(s) from mobile device/tablet here

____

Does a visual inspection of the habitat indicate the presence of larvae and/or pupae?	◯ Yes<br>◯ No
What did you use to sample the habitat?	◯ Dipper<br>◯ Long-handled dipper
Number of dips taken from habitat?	______________________

PART VI: SumiLarv 2MR Monitoring

Does the habitat have SumiLarv 2MR discs present?	◯ No<br>◯ Yes<br>◯ Not applicable
How many discs are present?	______________________
Do the 2MR discs have algae on them?	◯ No<br>◯ Yes
What condition are the 2MR discs in?	◯ Clean<br>◯ Slightly dirty<br>◯ Very dirty

PART VII: Field worker identification

Field worker name or initials	______________________
Supervisor name or initials	______________________
