# Supplementary material for: Field evaluation of SumiLarv™ 2MR larvicide for controlling the invasive malaria vector Anopheles stephensi in Ethiopia: a randomised controlled trial
Source: Malar J. 2026 Aug 17;25:314. doi: 10.1186/s12936-026-06089-3 (PMC13531892; doi:10.1186/s12936-026-06089-3)
Supplement: Supplementary file 2 [file 12936_2026_6089_MOESM2_ESM.docx]

SUMILARV KAAGA BIRKAAGA
ADIGA IYO QOYSKAAGABA KA ILAALI DUUMADA

WAA MAXAY SUMILARV?

SumiLarv waa saxan caag ah oo ay ku jirto kiimiko ka joojisa kaneecada aan qaan-gaarin inay u koraan kaneecada qaangaarka ah.

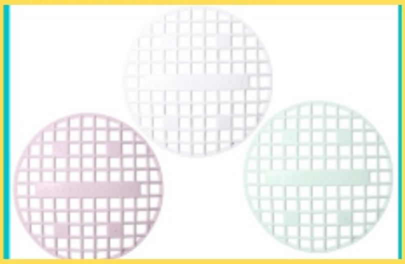

SIDEE SUMILARV KU ILAALIYAA IYO QOYSKAAGA DUUMADA

GiKaneecadu waxay ukunta biyaha ku shubtaa weelasha ay ka mid yihiin beesha birka. Ukumaha u koraan kaneecada dadka waaweyn kaasoo gudbin kara duumada. By ka hortagga kaneecada dadka waaweyn ka soo koraya, SumiLarv waxay dhimaysaa tirade kaneecada gudbisa duumada beeshaada

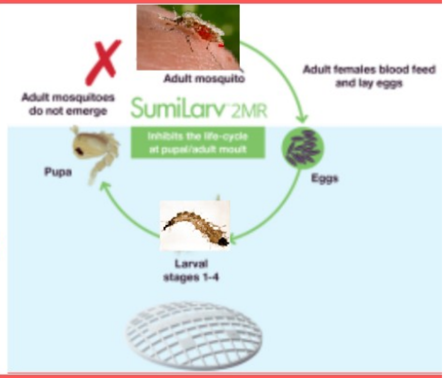

SIDA LOO ISTICMAALO SUMILARV DISCS?

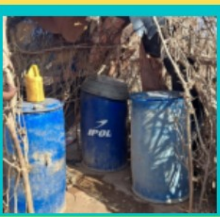

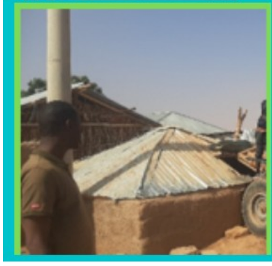

Saxanka waxaa lagu dhejin karaa biyo kasta

Weel laga heli karo gurigaaga iyo bulshada, tusaale ahaan foosto, durbaanno, biyo sare taangiyada ama birkadaha.

Saxanka ayaa hoos u degi doona ee weelasha. Marka dhowr saxanadaha waxa lagu daraa weel waa la isku xidhi karaa xadhig.

WAA BIYO LAGU DAAWAYNAYAA SUMILARV BADBAADO U AH CABITAANKA?

Haa, SumiLarv aad buu ammaan u yahay. Biyaha weelasha lagu daweeyaa SumiLarv waxay badbaado u tahay cabitaanka, karinta, qubayska, ku dhaqida guryaha iyo adigaba xoolaha.

IMMISA AYAY JIRTAA SUMILARV?

Saxanadaha SumiLarv waxay si fiican u shaqeeyaan ilaa 6 bilood.

MARKAAD ISTICMAALAYSO SUMILARV:

- Ha ka saarin saxanadaha weelasha ama birkas
- Ha u oggolaan carruurtu inay ku ciyaaraan saxanadaha
- Haddii aad u baahan tahay inaad weelka dhaqdo, si taxadar leh ka saar saxanka oo ka ilaali qorraxda.
- Ha iloobin inaad ku darto saxanka weel marka aad dhammayso dhaqid!
- Ha la wadaagin saxanadaha guryaha kale kuwaas oo aan haysan saxanadaha

WARBIXIN DHEERAAD AH

La xidhiidh:
Professor Delenesaw Yewhalaw(PhD.)
Taleefanka: +251917804352
Email:delenasawye@yahoo.com
Alemayehu Dagne
Taleefanka: +251 91 389 6700
Email: alemayehudagne@gmail.com
