# Supplementary material for: Field evaluation of SumiLarv™ 2MR larvicide for controlling the invasive malaria vector Anopheles stephensi in Ethiopia: a randomised controlled trial
Source: Malar J. 2026 Aug 17;25:314. doi: 10.1186/s12936-026-06089-3 (PMC13531892; doi:10.1186/s12936-026-06089-3)
Supplement: Supplementary file 2 [file 12936_2026_6089_MOESM10_ESM.docx]

**Supplementary file 9: Photographs of SumiLarv^TM^ 2MR**  **disc samples**

**Photo 1: Sumilarv 2MR samples (ID 02013)**

**Photo 2: Sumilarv 2MR samples (ID 02057)**

**Photo 3: Sumilarv 2MR samples (ID 02358)**

**Photo 4: Sumilarv 2MR samples (ID 02231)**
