# Supplementary material for: Field evaluation of SumiLarv™ 2MR larvicide for controlling the invasive malaria vector Anopheles stephensi in Ethiopia: a randomised controlled trial
Source: Malar J. 2026 Aug 17;25:314. doi: 10.1186/s12936-026-06089-3 (PMC13531892; doi:10.1186/s12936-026-06089-3)
Supplement: Supplementary file 3 [file 12936_2026_6089_MOESM11_ESM.docx]

Supplementary file 10: The coefficient of variation (%CV) and percentage release for the sub-sample of used Sumilarv ^TM^ 2MR.

| **Sample ID** | **Label** | **Remaining PPF %** | **% Release rate** | **AVG** | **SD** | **%CV** |
| --- | --- | --- | --- | --- | --- | --- |
| ID 02201 | M | 0.20 | 89.90 | 0.23 | 0.13 | 56.52 |
|  | end1 | 0.12 | 93.94 |  |  |  |
|  | end2 | 0.37 | 81.31 |  |  |  |
| ID 02026 | M | 0.10 | 94.95 | 0.11 | 0.01 | 11.00 |
|  | end1 | 0.12 | 93.94 |  |  |  |
|  | end2 | 0.10 | 94.95 |  |  |  |
| ID 02147 | M | 0.44 | 77.78 | 0.41 | 0.03 | 7.47 |
|  | end1 | 0.38 | 80.81 |  |  |  |
|  | end2 | 0.41 | 79.29 |  |  |  |
| ID 02204 | M | 0.20 | 89.90 | 0.17 | 0.03 | 17.97 |
|  | end1 | 0.16 | 92.92 |  |  |  |
|  | end2 | 0.14 | 92.93 |  |  |  |
| ID 02040 | M | 0.52 | 73.74 | 0.54 | 0.02 | 4.09 |
|  | end1 | 0.53 | 73.23 |  |  |  |
|  | end2 | 0.56 | 71.72 |  |  |  |
| ID 02231 | M | 0.10 | 94.95 | 0.11 | 0.01 | 10.51 |
|  | end1 | 0.12 | 93.94 |  |  |  |
|  | end2 | 0.10 | 94.95 |  |  |  |
| ID 02103 | M | 0.64 | 67.68 | 0.51 | 0.26 | 51.34 |
|  | end1 | 0.68 | 65.65 |  |  |  |
|  | end2 | 0.21 | 89.39 |  |  |  |
| ID 02268 | M | 0.41 | 79.29 | 0.35 | 0.06 | 16.46 |
|  | end1 | 0.30 | 84.85 |  |  |  |
|  | end2 | 0.34 | 82.83 |  |  |  |
| ID 02057 | M | 0.04 | 97.98 | 0.04 | 0.00 | 6.12 |
|  | end1 | 0.04 | 97.98 |  |  |  |
|  | end2 | 0.04 | 97.98 |  |  |  |
| ID 02060 | M | 0.37 | 81.31 | 0.39 | 0.03 | 8.47 |
|  | end1 | 0.43 | 78.28 |  |  |  |
|  | end2 | 0.36 | 81.82 |  |  |  |
| ID 02050 | M | 0.59 | 70.20 | 0.61 | 0.02 | 3.39 |
|  | end1 | 0.60 | 69.70 |  |  |  |
|  | end2 | 0.63 | 68.18 |  |  |  |
| ID 02231 | M | 0.60 | 69.70 | 0.58 | 0.02 | 4.24 |
|  | end1 | 0.55 | 72.22 |  |  |  |
|  | end2 | 0.58 | 70.71 |  |  |  |
| ID 02013 | M | 0.65 | 67.17 | 0.61 | 0.03 | 5.66 |
|  | end1 | 0.58 | 70.71 |  |  |  |
|  | end2 | 0.60 | 69.70 |  |  |  |
| ID 02275 | M | 0.49 | 75.25 | 0.42 | 0.07 | 17.55 |
|  | end1 | 0.35 | 82.32 |  |  |  |
|  | end2 | 0.40 | 79.80 |  |  |  |
| ID 02336 | M | 0.10 | 94.95 | 0.17 | 0.10 | 59.23 |
|  | end1 | 0.28 | 85.86 |  |  |  |
|  | end2 | 0.12 | 93.94 |  |  |  |
| ID 02358 | M | 0.52 | 73.74 | 0.46 | 0.06 | 12.61 |
|  | end1 | 0.41 | 79.29 |  |  |  |
|  | end2 | 0.45 | 77.27 |  |  |  |

**For 95% CI = 79.13% – 85.04%**

**Point estimate = (79.13-85.04)/2=82.085**

**Uncertainty = (85.04-79.13)/2=2.955 %**

**Uncertainty = ± 2.96% (rounded to ± 3.0%)**

| **Descriptives** | | | | | |
| --- | --- | --- | --- | --- | --- |
|  | code | | | Statistic | Std. Error |
| %release | 1 | Mean | | 82.081228956228900 | 1.469310168791483 |
|  |  | 95% Confidence Interval for Mean | Lower Bound | 79.125358162436440 |  |
|  |  |  | Upper Bound | 85.037099750021350 |  |
|  |  | 5% Trimmed Mean | | 82.056677890011170 |  |
|  |  | Median | | 81.060606060606050 |  |
|  |  | Variance | | 103.626 |  |
|  |  | Std. Deviation | | 10.179679457697802 |  |
|  |  | Minimum | | 65.6565656565656 |  |
|  |  | Maximum | | 97.9797979797979 |  |
|  |  | Range | | 32.3232323232323 |  |
|  |  | Interquartile Range | | 21.2121212121212 |  |
|  |  | Skewness | | .099 | .343 |
|  |  | Kurtosis | | -1.381 | .674 |
