# Supplementary material for: Field evaluation of SumiLarv™ 2MR larvicide for controlling the invasive malaria vector Anopheles stephensi in Ethiopia: a randomised controlled trial
Source: Malar J. 2026 Aug 17;25:314. doi: 10.1186/s12936-026-06089-3 (PMC13531892; doi:10.1186/s12936-026-06089-3)
Supplement: Supplementary file 4 [file 12936_2026_6089_MOESM1_ESM.docx]

**Supplementary files:**

**Supplementary file 1:** Informative communication material (Poster) in local language.

**Supplementary file 2:** Longitudinal habitat census and characterization form

**Supplementary file 3:** Methodology of pyriproxyfen extraction from discs

**Supplementary file 4:** Container capacity and application of SumiLarv^TM^ 2MR discs

**Supplementary file 5:** Mean number of SumiLarv^TM^ 2MR discs in treatment *birkas* across time points

**Supplementary file 6:** Summary of the generalized linear mixed model (GLMM)

**Supplementary file 7:** Amplification plots for *An. stephensi* samples from control *birkas* (A) and treated *birkas* (B)

**Supplementary file 8:** Analysis of the remaining concentration of PPF in SumiLarv^TM^ 2MR discs

**Supplementary file 9:** Photographs of SumiLarv^TM^ 2MR disc samples
