# Supplementary material for: Field evaluation of SumiLarv™ 2MR larvicide for controlling the invasive malaria vector Anopheles stephensi in Ethiopia: a randomised controlled trial
Source: Malar J. 2026 Aug 17;25:314. doi: 10.1186/s12936-026-06089-3 (PMC13531892; doi:10.1186/s12936-026-06089-3)
Supplement: Supplementary file 6 [file 12936_2026_6089_MOESM4_ESM.docx]

The 82.1 % release and 17.67 % remaining PPF are calculated by comparing the field sample average (0.355 w/w%) to the fresh sample average (1.98 w/w%), treating the fresh value as the initial reference after any minor deviation from the 2.0% label.

## Calculation Method

Subtract the field content from the fresh content, then divide by the fresh content for the released fraction:
((1.98 - 0.35) / 1.98) × 100 = 82.10% released.
The remaining fraction is simply (0.35 / 1.98) × 100 = 17.67%.

## Context in Vector Control

This quantifies PPF release from Sumilarv 2MR discs (a slow-release larvicide for Anopheles mosquitoes) under field conditions in Ethiopia, where ~82.1% elution over time leaves 17.67% residual active ingredient.

**Supplementary file 3: Methodology of pyriproxyfen extraction from discs SumiLarv™ 2MR** **discs**

**Methodology**

The analysis was conducted in accordance with the published CIPAC method (715 / MR / M / 3, CIPAC / 4997m, CIPAC Handbook P, p.177, 2021) for determining the active ingredient (pyriproxyfen) content in SumiLarv™ 2MR.

**1- Materials Used**
Pyriproxyfen T.G. (Sumitomo chemical Co., Ltd.), Lot No.120109, Purity 99.2%
Dicyclohexyl Phthalate (Sigma-Aldrich), Lot N0. 08122TVD
Ethyl acetate (Loba Chemie PVT Ltd), Lot N0. B350522106
SumiLarv™ 2MR unused samples (Sumitomo Chemical Co. Ltd.), Lot No. 2X01MRG
SumiLarv™ 2MR used in CEASE project trial (Sumitomo Chemical Co. Ltd.), Lot No. 1Z01MRW
**2- Preparation of Standard Solutions**

**Internal Standard Solution**: A 2.50 g sample of dicyclohexyl phthalate was accurately weighed and dissolved in 200 mL of ethyl acetate to prepare the internal standard solution. This solution was further diluted tenfold to make a sufficient volume for sample extraction.

**Pyriproxyfen Calibration Standard Solution:** Pyriproxyfen calibration standards (CA & CB) were prepared in duplicate by accurately weighing between 45 mg to 55 mg of pyriproxyfen and dissolving it in 90.0 mL of ethyl acetate. An aliquot of 10.0 mL of the internal standard solution was then added to each calibration solution. To assess the linearity of the analytical method, three different concentrations of pyriproxyfen standards (0.5, 1.0, and 2.0 mg/mL) were prepared.

**3- Extraction of pyriproxyfen from SumiLarv™ 2MR** **for analysis**

**Unused Samples**: A SumiLarv™ 2MR unused disc was cut into small pieces, approximately 0.5 g was weighed, and transferred into 20 mL glass vials. A 10.0 mL aliquot of the dicyclohexyl phthalate internal standard solution was added to each vial. The mixture was shaken and left at room temperature for 24 hours. After this extraction period, the sample solution was filtered using a membrane filter and analyzed by high-performance liquid chromatography (HPLC) at the ATRC Analytical Unit, Arusha.

**Samples from Ethiopia**: Similarly, the SumiLarv™ 2MR samples from Ethiopia were processed in the same manner. Approximately 1.0 g of the sample was cut into small pieces, and 10.0 mL of the dicyclohexyl phthalate internal standard solution was added. The extraction was carried out at room temperature for 24 hours, followed by filtration and HPLC analysis. The analysis was only initiated after confirming that the method was functioning correctly with the ATRC unused samples.

**4- HPLC analysis**

The linearity solutions were initially injected to assess the linearity of the analytical method, followed by the calibration solutions. Sample injections were performed in duplicate, with each sample being bracketed by the calibration solutions in the following sequence: CA, S1, S1, CB, S2, S2, CA, S3, S3, CB, and so on.

**HPLC conditions for Pyriproxyfen analysis**

HPLC: 1260 Infinity Series

Detection Wavelength: 254 nm

Column Temperature: 40 oC

Injection Volume: 5 μL

Flow Rate: 1.2 mL/min

Mobile Phase: Acetonitrile: Water, (2:1 v/v)

Column: Zobrax Eclipse Plus C18 150 mm X 4.6 mm, 3.5 μM
