# Supplementary material for: Field evaluation of SumiLarv™ 2MR larvicide for controlling the invasive malaria vector Anopheles stephensi in Ethiopia: a randomised controlled trial
Source: Malar J. 2026 Aug 17;25:314. doi: 10.1186/s12936-026-06089-3 (PMC13531892; doi:10.1186/s12936-026-06089-3)
Supplement: Supplementary file 7 [file 12936_2026_6089_MOESM5_ESM.docx]

**Containers capacity and application of SumiLarv^TM^ 2MR discs (supplementary file 4)**

| **container ID** | **length** | **width** | **depth** | **container capacity** | **Number of SumiLarv 2MR discs** |
| --- | --- | --- | --- | --- | --- |
| 2013 | 3.5 | 3.6 | 2.25 | 28350 | 106.3125 |
| 2017 | 3.6 | 3.7 | 2.02 | 26906.4 | 100.899 |
| 2026 | 3.6 | 3.62 | 1.8 | 23457.6 | 87.966 |
| 2035 | 3.3 | 3.6 | 2.25 | 26730 | 100.2375 |
| 2040 | 3.2 | 3.4 | 2.6 | 28288 | 106.08 |
| 2045 | 3.5 | 3.5 | 2.1 | 25725 | 96.46875 |
| 2050 | 3.7 | 3.7 | 2.15 | 29433.5 | 110.375625 |
| 2051 | 3.8 | 3.8 | 1.96 | 28302.4 | 106.134 |
| 2057 | 3.3 | 3.5 | 2.1 | 24255 | 90.95625 |
| 2060 | 3.4 | 3.45 | 2.04 | 23929.2 | 89.7345 |
| 2067 | 3.6 | 3.7 | 2.15 | 28638 | 107.3925 |
| 2072 | 3.4 | 3.6 | 2.2 | 26928 | 100.98 |
| 2078 | 3.6 | 3.6 | 2.09 | 27086.4 | 101.574 |
| 2083 | 3.5 | 3.6 | 2.03 | 25578 | 95.9175 |
| 2089 | 3.4 | 3.5 | 2.1 | 24990 | 93.7125 |
| 2091 | 3.6 | 3.7 | 2 | 26640 | 99.9 |
| 2097 | 3.6 | 3.6 | 2.01 | 26049.6 | 97.686 |
| 2103 | 3.6 | 3.7 | 2.1 | 27972 | 104.895 |
| 2110 | 3.6 | 3.6 | 2.1 | 27216 | 102.06 |
| 2135 | 3.65 | 3.65 | 2 | 26645 | 99.91875 |
| 2147 | 3.8 | 3.85 | 2 | 29260 | 109.725 |
| 2154 | 3.4 | 3.5 | 2.15 | 25585 | 95.94375 |
| 2176 | 3.6 | 3.6 | 2.1 | 27216 | 102.06 |
| 2182 | 3.6 | 3.7 | 2 | 26640 | 99.9 |
| 2201 | 3.72 | 3.72 | 2.05 | 28368.72 | 106.3827 |
| 2204 | 3.7 | 3.7 | 2.1 | 28749 | 107.80875 |
| 2215 | 3.3 | 3.56 | 2.15 | 25258.2 | 94.71825 |
| 2230 | 3.8 | 3.9 | 1.8 | 26676 | 100.035 |
| 2231 | 3.2 | 3.3 | 2 | 21120 | 79.2 |
| 2244 | 3.6 | 3.6 | 2.067 | 26788.32 | 100.4562 |
| 2248 | 3.6 | 3.7 | 1.85 | 24642 | 92.4075 |
| 2268 | 3.65 | 3.65 | 2.1 | 27977.25 | 104.9146875 |
| 2275 | 3.72 | 3.76 | 2.17 | 30352.224 | 113.82084 |
| 2281 | 3.65 | 3.65 | 2.1 | 27977.25 | 104.9146875 |
| 2282 | 3.55 | 3.67 | 2.15 | 28011.275 | 105.0422813 |
| 2289 | 3.6 | 3.7 | 2.1 | 27972 | 104.895 |
| 2293 | 3.62 | 3.67 | 2.1 | 27899.34 | 104.622525 |
| 2308 | 3.7 | 3.7 | 2.04 | 27927.6 | 104.7285 |
| 2311 | 3.4 | 3.5 | 2.28 | 27132 | 101.745 |
| 2320 | 3.6 | 3.7 | 2.1 | 27972 | 104.895 |
| 2328 | 3.6 | 3.6 | 2.02 | 26179.2 | 98.172 |
| 2331 | 3.6 | 3.7 | 2.1 | 27972 | 104.895 |
| 2332 | 3.38 | 3.7 | 2.18 | 27263.08 | 102.23655 |
| 2336 | 3.6 | 3.65 | 2.11 | 27725.4 | 103.97025 |
| 2358 | 3.25 | 3.3 | 2.06 | 22093.5 | 82.850625 |
| 2366 | 3.46 | 3.65 | 1.73 | 21848.17 | 81.9306375 |
| 2397 | 3.6 | 3.6 | 2.02 | 26179.2 | 98.172 |
| 2406 | 3.37 | 3.6 | 2.1 | 25477.2 | 95.5395 |
| 2407 | 3.5 | 3.6 | 2.1 | 26460 | 99.225 |
| 2408 | 3.4 | 3.54 | 2.1 | 25275.6 | 94.7835 |
| 2410 | 2.8 | 4 | 2.2 | 24640 | 92.4 |
