# Supplementary material for: Field evaluation of SumiLarv™ 2MR larvicide for controlling the invasive malaria vector Anopheles stephensi in Ethiopia: a randomised controlled trial
Source: Malar J. 2026 Aug 17;25:314. doi: 10.1186/s12936-026-06089-3 (PMC13531892; doi:10.1186/s12936-026-06089-3)
Supplement: Supplementary file 9 [file 12936_2026_6089_MOESM7_ESM.docx]

**S6: Summary of the generalized linear mixed model (GLMM)**

This is the summary of a generalized linear mixed model (GLMM) fitted with a negative binomial distribution (nbinom2) and a log link function.

**Model Overview**

- **Response Variable:** N_pupae_ano ( number of pupae)
- **Fixed Effect Predictor:** type_habitat ( control or treatment containers)
- **Random Effect:** Habitat.ID (individual containers)
- **Distribution:** Negative binomial (nbinom2)
- **Log Link Function:** The model estimates log-transformed counts

**Random Effects (Variation Across Habitat.ID)**

- **Variance:** 1.039
- **Standard Deviation:** 1.019
  - There is some variation in N_pupae_ano between different Habitat.IDs.

**Fixed Effects (Predictor Variables)**

|  | Estimate | Standard error | Z-value | p-value |
| --- | --- | --- | --- | --- |
| Intercept | 0.111 | 0.149 | 0.745 | 0.457 |
| Type_habitat  (Intervention vs control ) | 0.044 | 0.206 | 0.212 | 0.832 |
