# Supplementary material for: Field evaluation of SumiLarv™ 2MR larvicide for controlling the invasive malaria vector Anopheles stephensi in Ethiopia: a randomised controlled trial
Source: Malar J. 2026 Aug 17;25:314. doi: 10.1186/s12936-026-06089-3 (PMC13531892; doi:10.1186/s12936-026-06089-3)
Supplement: Supplementary file 11 [file 12936_2026_6089_MOESM9_ESM.docx]

**Chromatogram 1 : Extraction solvent (Ethyl Acetate)**

**Chromatogram 2: PPF Calibration solution ( 0.5mg/mL)**

**Chromatogram 3: Sumilarv^TM^ 2MR unused sample solution from ATRC**

**Chromatogram 4: Sumilarv^TM^ 2MR unused sample solution from ATRC**

**Supplementary file 8:** Analysis of the remaining concentration of PPF in Sumilarv^TM^ 2MR
