# Supplementary material for: Field evaluation of SumiLarv™ 2MR larvicide for controlling the invasive malaria vector Anopheles stephensi in Ethiopia: a randomised controlled trial
Source: Malar J. 2026 Aug 17;25:314. doi: 10.1186/s12936-026-06089-3 (PMC13531892; doi:10.1186/s12936-026-06089-3)
Supplement: Supplementary file 13 [file 12936_2026_6089_MOESM6_ESM.docx]

**S5:**  Mean number of Sumilarv 2 MR discs in treatment *birkas* across time points
